# Discordant Treatment Responses to Combination Antiretroviral Therapy in Rwanda: A Prospective Cohort Study

**DOI:** 10.1371/journal.pone.0159446

**Published:** 2016-07-20

**Authors:** Felix R. Kayigamba, Molly F. Franke, Mirjam I. Bakker, Carly A. Rodriguez, Emmanuel Bagiruwigize, Ferdinand WNM Wit, Michael L. Rich, Maarten F. Schim van der Loeff

**Affiliations:** 1 INTERACT, CPCD, PO Box 2181, Kigali, Rwanda; 2 Department of Global Health and Social Medicine, Harvard Medical School, Boston, MA, United States of America; 3 Partners In Health/Inshuti Mu Buzima, Rwinkwavu, Rwanda; 4 Royal Tropical Institute, KIT Biomedical Research, Amsterdam, the Netherlands; 5 Amsterdam Institute for Global Health and Development (AIGHD), Amsterdam, the Netherlands; 6 Center for Infection and Immunity Amsterdam (CINIMA), Academic Medical Center (AMC), Amsterdam, the Netherlands; 7 Division of Global Health Equity, Brigham and Women’s Hospital, Boston, MA, United States of America; 8 Public Health Service of Amsterdam (GGD), Amsterdam, the Netherlands; Centro de Biología Molecular Severo Ochoa (CSIC-UAM), SPAIN

## Abstract

**Introduction:**

Some antiretroviral therapy naïve patients starting combination antiretroviral therapy (cART) experience a limited CD4 count rise despite virological suppression, or vice versa. We assessed the prevalence and determinants of discordant treatment responses in a Rwandan cohort.

**Methods:**

A discordant immunological cART response was defined as an increase of <100 CD4 cells/mm^3^ at 12 months compared to baseline despite virological suppression (viral load [VL] <40 copies/mL). A discordant virological cART response was defined as detectable VL at 12 months with an increase in CD4 count ≥100 cells/mm^3^. The prevalence of, and independent predictors for these two types of discordant responses were analysed in two cohorts nested in a 12-month prospective study of cART-naïve HIV patients treated at nine rural health facilities in two regions in Rwanda.

**Results:**

Among 382 patients with an undetectable VL at 12 months, 112 (29%) had a CD4 rise of <100 cells/mm^3^. Age ≥35 years and longer travel to the clinic were independent determinants of an immunological discordant response, but sex, baseline CD4 count, body mass index and WHO HIV clinical stage were not. Among 326 patients with a CD4 rise of ≥100 cells/mm^3^, 56 (17%) had a detectable viral load at 12 months. Male sex was associated with a virological discordant treatment response (*P* = 0.05), but age, baseline CD4 count, BMI, WHO HIV clinical stage, and travel time to the clinic were not.

**Conclusions:**

Discordant treatment responses were common in cART-naïve HIV patients in Rwanda. Small CD4 increases could be misinterpreted as a (virological) treatment failure and lead to unnecessary treatment changes.

## Introduction

The aim of combination antiretroviral therapy (cART) is to suppress plasma human immunodeficiency virus (HIV) viral load (VL) to undetectable levels. The usual median time to achieve full viral suppression is about 100 days [1,2]. Most HIV patients, both in high-income and in resource-poor countries, also display an immunological response to treatment, measured as an increase in CD4 count.[3–5] In 14–25% of patients CD4 count does not rise substantially despite successful viral suppression.[1,6–9] This phenomenon has been referred to as an immunological discordant treatment response.

Studies have reported an increased incidence of AIDS events or death among those with immunological discordant responses.[1,6,8–11] The mortality risk among immunological discordant responders is between that of complete responders and that of complete non-responders, [6,8] thus, discordant treatment responses are regarded as suboptimal treatment outcomes.

Older age and lower baseline VL have consistently been shown to be associated with discordant response.[1,6,7,10,12–14] Low adherence and lamivudine or zidovudine containing regimens were also found to be associated with a discordant response.[6] Studies examining the relationship between baseline CD4 cell count and discordant response show conflicting results, with some reporting a positive association between low CD4 count and a discordant treatment response,[1,6,15] and others the reverse.[7,10] Most studies on discordant responses have been done in cohorts from high-income countries.

Another type of discordant treatment response is a positive immunological response despite incomplete suppression of viral replication. This type of response is was found to be associated with a history of injecting drug use, high baseline HIV VL, and poor adherence.[6] Those with a discordant virological response have a higher mortality risk,[6,8,9,11] and like discordant immunological responses, is regarded as a suboptimal treatment response.

In practice, routine viral load monitoring is recommended to detect treatment failure earlier and accurately;[16] however, in resource-limited settings where routine virological monitoring is not available, immunological and clinical criteria are often used. As a result, patients presenting with a negative immunological response may be misclassified as having failed treatment, and unnecessarily switched to costly second-line regimens. For this reason, understanding discordant treatment responses, and the factors that influence them, is critical to optimizing cART use. We studied the frequency of discordant treatment responses in a cohort of cART-naïve HIV patients starting cART in Rwanda, and assessed determinants of discordant responses in this setting.

## Methods

Rwanda is one of only three countries in sub-Saharan Africa with a generalised HIV epidemic where over 90% of ART eligible HIV patients are on cART.[17] A dense network of clinics and hospitals provide HIV care and treatment, free of cost.[18] From a prospective study of 610 ART-naïve HIV infected patients starting cART at nine health facilities in Rwanda,[19] we identified two nested cohorts of patients for analyses of discordant immunological and virological response.

A detailed description of the study methods and of treatment outcomes of the full cohort has been published.[19] In brief, patient enrollment started in June 2007 and ended in August 2008. Inclusion criteria were: (1) documented HIV infection; (2) starting cART at one of the nine selected Ministry of Health (MOH) centers in the two study regions; (3) residence in one of the study regions for at least the past one year. Patients were excluded if CD4 count was above 350 cells/mm^3^ at the time of cART initiation, if they were aged less than 21 years or if they had previously initiated cART (except for women who had received short-term antiretrovirals during pregnancy).

### Standard of care for cART

cART was provided free-of-charge to all individuals who met eligibility criteria based on Rwandan Ministry of Health guidelines.[20] In short, eligibility criteria for cART at the time of the study included: confirmed HIV seropositivity; WHO clinical stage 4 regardless of CD4 count or WHO clinical stage 1, 2, or 3 with a CD4 count of <350/mm^3^; and fulfillment of mandatory social conditions. Current guidelines call for consideration of regimen switches when there is suspicion of clinical, immunological or virological treatment failure, and only after careful assessment of adherence and repeat CD4 cell count and/or viral load testing. The first-line cART regimen for HIV-infected individuals consisted of either stavudine or zidovudine, plus lamivudine and nevirapine. Efavirenz replaced nevirapine in individuals who were receiving tuberculosis (TB) treatment. Co-trimoxazole was routinely prescribed to individuals with CD4 cell counts <350 cells/mm^3^ or World Health Organization (WHO) HIV clinical stage 3 or 4.[21] CD4 cell counts were routinely measured prior to initiation of cART. All patients were urged to disclose their HIV status to at least one family member or friend and identify a “treatment buddy” in order to facilitate treatment adherence. At five of the nine health centers, patients additionally received community-based social support from community health workers (including directly observed treatment), nutritional support, transportation stipends, and other support as needed.[19]

### Data collection

A baseline clinical exam was done before cART initiation for all patients enrolled into the study. A standardized intake form was completed regarding age, sex, marital status, literacy, study site, patient travel time to the clinic, weight and height, CD4 cell count, WHO clinical stage, cART start date, antiretroviral regimen, and whether the patient was being treated for TB at the time of initiation of cART. CD4 cell count measurements were done at baseline and after 12 months, using the FACS Count system (Becton Dickinson TM, La Pont de Claix, France). Plasma VL measurement was done only after 12 months of cART using the Cobas TaqMan 48 Analyzer (Roche, Geneva, Switzerland); the threshold level for detection of VL was 40 copies/mL. During monthly clinic visits patients were examined and any diagnosed opportunistic infections were treated. No study participant was prescribed a second-line cART regimen during their first year on cART (i.e., their study follow up period). Adherence was assessed 3 months and 12 months after the start of cART, using the validated Center for Adherence Support Evaluation (CASE) adherence index. This index is a simple composite measure of self-reported ART adherence, based on three questions.[22] The theoretical score range is from 3 to 16, however pilot data suggested that participants had difficulty distinguishing between two response categories: missing a dose an average of “zero times per week” and “less than once a week”. We therefore combined these response categories for a total maximum score of 15. A CASE index score of ≤10 indicates suboptimal adherence.[22]

### Selection of Nested Cohorts

**Fig 1:**
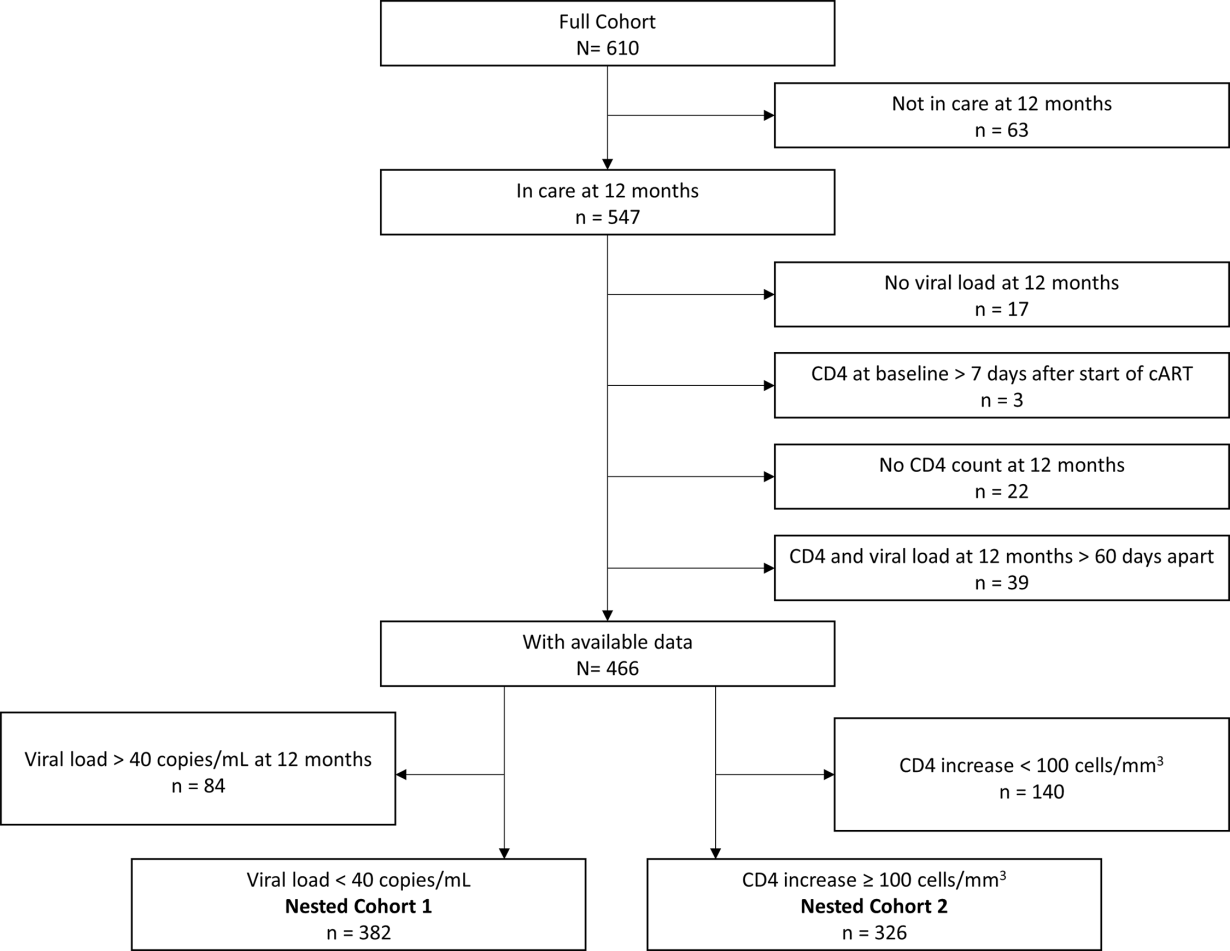
Flow chart showing how Nested Cohort 1 and Nested Cohort 2 were selected from the full cohort, Rwanda, 2008–2009.

Of the 610 patients included in the full cohort, 35 (6%) died by the end of the 12-month observation period, 13 (2%) defaulted, 15 (3%) were transferred to other clinics, and 547 patients were retained in care at 12 months (Fig 1). From 17 (3%) of these 547 patients, no VL measurement was available; the baseline CD4 count was done > 7 days after start of cART in 3 patients (1%); from 22 (4%) no CD4 count was available at the 12-month time point; and from 39 patients (7%) the dates of the end-of-observation period CD4 count and VL measurement were >60 days apart. Thus, essential measurements were available for 466 patients still in care at 12 months.

**Nested Cohort 1** consisted of patients who had undetectable VL at 12 months (n = 382). Nested Cohort 1 allowed us to study what is referred to as “immunological cART discordance”. This was defined as an increase of <100 CD4 cells/mm^3^ at 12 months compared to baseline in spite of full virological suppression (VL<40 copies/mL).

**Nested Cohort 2** consisted of patients enrolled in the prospective study who had experienced an increase in CD4 count of ≥100 cells/mm^3^ at 12 months compared to baseline (n = 326). Nested Cohort 2 allowed us to examine “virological cART discordance”, defined as an increase of CD4 count of ≥100 cells/mm^3^ in the first 12 months and incomplete viral suppression at month 12.

Of note, the definitions used to define suboptimal immunological (increase of <100 CD4 cells/mm^3^) and virological responses (VL≥40 copies/mL) at twelve months were intentionally broad, relative to WHO definitions of immunological and virological treatment failure,[23] in order to encompass the majority of patients at risk of either type of treatment failure.

### Statistical analysis

Bivariate logistic regression analysis was done to identify factors that were associated with discordant treatment responses. Subsequently, multivariable logistic regression was performed to identify independent determinants of a discordant treatment response. The variables sex, age, CD4 count at baseline and region were retained in the model irrespective of P values. Other variables were included into a starting model if they were associated with the outcome at *P*<0.20 in bivariate analysis. These variables were dropped one by one, based on a criterion of *P*<0.05, using the likelihood ratio test, until a parsimonious model was obtained.

In a secondary analysis, we examined whether adherence to cART, assessed 3 and 12-months (+/- 1 month) after the start of cART, was predictive of discordant treatment responses.

All reported *P* values were two-sided. *P* values <0.05 were considered statistically significant. All analyses were done using Stata 11 (StataCorp, College Station, Texas, USA).

### Ethical approval

The study protocol was approved by the Rwanda National Ethics Committee Kigali, Rwanda and the Partners Human Research Committee, Boston, USA. All individual patients recruited into the study signed consent forms before they were enrolled. Data were double entered into an electronic medical record system (OpenMRS) that was password-protected; patient identification codes instead of names were used during analysis to ensure the confidentiality.

## Results

Of the 466 patients who were retained in care after 12 months and who had a 12-month VL and CD4 count measurement done, 140 had a CD4 rise <100 cells/mm^3^ (Table 1). Of these, 28 had a detectable VL while 112 had full viral suppression (i.e., a discordant immunological response). Only seven of those 28 had a VL of 1,000 copies/mL or above. Thus, of patients identified with a limited CD4 rise after 12 months, only 5% (7/140; 95%CI 2–10%) had virological failure (here defined as a single VL ≥1,000 copies/mL). With regard to virological discordance, 84 of 466 patients had a viral load ≥ 40 copies/mL at month 12, of whom 56 experienced a CD4 count increase of ≥100 cells/mm^3^.

**Table 1:** Virological and immunological treatment responses among 466 patients retained in care at 12 months and with full data, Rwanda, 2007–2008.

		Viral Load		
		<40 copies/mL = Nested Cohort 1	≥40 copies/mL	All
CD4 increase	<100 cells/mm^3^	112	28	140
	≥100 cells/mm^3^ = Nested Cohort 2	270	56	326
	All	382	84	466

### Analysis of immunological discordant treatment responses: Nested Cohort 1

Table 2 shows the baseline characteristics of Nested Cohort 1, alongside the characteristics of the full cohort. The median age (interquartile range [IQR]) was 40 (34–47) years and 64% of patients were female. The median (IQR) CD4 count at baseline was 240 (150–295) cells/mm^3^ and 46% (177/382) of patients were in WHO stage 3 or 4. The median time between baseline CD4 count and start of cART was 22 (9–41) days

**Table 2:** Baseline characteristics of Nested Cohorts 1 and 2 and of the full cohort of 610 HIV infected patients starting cART, Rwanda, 2007–2008.

	Full Cohort N = 610	Nested Cohort 1 (for the analysis of immunological discordant cART response) N = 382	Nested Cohort 2 (for the analysis of virological discordant cART response) N = 326
Age			
Median age (IQR) in years	40 (34–47)	40 (34–47)	39 (33–46)
20–34 years	159 (26.1%)	98 (25.7%)	101 (31.0%)
35–44 years	256 (42.0%)	159 (41.6%)	130 (39.9%)
≥45 years	195 (32.0%)	125 (32.7%)	95 (29.1%)
Sex			
Male	234 (38.4%)	139 (36.4%)	122 (37.4%)
Female	376 (61.6%)	243 (63.6%)	204 (62.6%)
Literacy			
Unable to read	203 (33.3%)	131 (34.3%)	113 (34.7%)
Able to read	407 (66.7%)	251 (65.7%)	213 (65.3%)
Marital status			
Single	28 (4.6%)	16 (4.2%)	16 (4.9%)
Married/cohabiting	351 (57.8%)	221 (58.9%)	193 (59.2%)
Divorced/separated	60 (9.9%)	38 (10.0%)	33 (10.1%)
Widowed	168 (27.7%)	107 (28.0%)	84 (25.8%)
Missing	3	--	--
CD4 count at baseline			
Median CD4 (IQR) cells/mm^3^	231 (148–289)	240 (150–295)	239 (155–294)
<100 cells/mm^3^	91 (14.9%)	53 (13.9%)	43 (13.2%)
100–199 cells/mm^3^	149 (24.4%)	82 (21.5%)	78 (23.9%)
200–350 cells/mm^3^	370 (60.7%)	247 (64.7%)	205 (62.9%)
Median time (IQR) in days CD4 count—start cART*	22 (9–41)	22 (9–41)	23 (9–42)
WHO Stage at baseline			
1	117 (19.2%)	73 (19.2%)	61 (18.8%)
2	196 (32.2%)	131 (34.4%)	115 (35.4%)
3	281 (46.1%)	168 (44.1%)	139 (42.8%)
4	15 (2.5%)	9 (2.4%)	10 (3.1%)
Missing	1	1	1
BMI at baseline in kg/m^2^			
Median BMI	20.7 (18.6–22.7)	20.7 (18.7–22.7)	20.8 (18.8–22.9)
<18.5	151 (25.0%)	87 (22.9%)	69 (21.3%)
18.5–24.9	398 (65.8%)	256 (67.2%)	223 (68.4%)
≥25	56 (9.3%)	37 (9.7%)	32 (9.8%)
Missing	5	2	2
On TB treatment at start of cART			
No	585 (96.2%)	366 (96.1%)	313 (96.3%)
Yes	23 (3.8%)	15 (3.9%)	12 (3.7%)
Missing	2	1	1
Regimen with d4T			
No	191 (31.3%)	117 (30.9%)	95 (29.1%)
Yes	416 (68.5%)	264 (69.3%)	229 (70.7%)
Missing	3	1	2
Regimen with NVP			
No	56 (9.2%)	34 (8.9%)	30 (9.2%)
Yes	551 (90.8%)	347 (91.1%)	294 (90.7%)
Missing	3	1	2
Travel time to clinic			
<30 minutes	129 (21.4%)	78 (20.5%)	62 (19.1%)
Between 30 & 60 minutes	148 (24.5%)	93 (24.4%)	83 (25.5%)
Between 1 & 2 hours	186 (30.8%)	124 (32.6%)	113 (34.8%)
> 2 hours	141 (23.3%)	86 (22.6%)	67 (20.6%)
Missing	6	1	1
Region			
Ruhengeri	306 (50.2%)	197 (51.6%)	163 (50.0%)
Kayonza/Kirehe	304 (49.8%)	185 (48.4%)	163 (50.0%)

Nested Cohorts 1 and 2 are not mutually exclusive cohorts, patients may be included in both. Numbers are n (%) or Median (IQR) unless indicated otherwise. HIV Human immunodeficiency virus; cART Combination antiretroviral treatment; BMI Body mass index; IQR Inter-quartile range; WHO World Health Organization; N Number; AZT zidovudine; 3TC lamivudine; d4T stavudine; NVP nevirapine; EFV efavirenz.

* Median time (IQR) in days between baseline CD4 count and date start of ART.

Table 3 shows the follow-up data of Nested Cohort 1, next to the data of the full cohort for comparison. The median (IQR) CD4 count at 12 months was 390 (179–502) cells/mm^3^ and the median increase between the baseline and the 12-month CD4 count was 163 (87–259) cells/mm^3^.

**Table 3:** Follow-up data of 610 HIV patients on cART, Rwanda, 2007–2008.

	Full cohort N = 610	Nested Cohort 1 N = 382	Nested Cohort 2 N = 326
CD4 count at 12 months			
Median CD4 (IQR) cells/mm^3^	392 (275–507)	390 (179–502)	454 (362–541)
<100 cells/mm^3^	11 (1.8%)	7 (1.8%)	--
100–199 cells/mm^3^	47 (9.0%)	32 (8.4%)	12 (3.7%)
200–349 cells/mm^3^	145 (27.7%)	110 (28.8%)	57 (17.5%)
350–499 cells/mm^3^	182 (34.8%)	134 (35.1%)	141 (43.3%)
≥500 cells/mm^3^	137 (26.2%)	99 (25.9%)	116 (35.6%)
Missing	88	--	
Difference in CD4 count between baseline and 12 months			
Median difference (IQR) cells/mm^3^	163 (85–258)	163 (87–259)	206 (156–297)
<0 cells/mm^3^	29 (4.8%)	20 (5.2%)	--
0–99 cells/mm^3^	127 (24.3%)	92 (24.1%)	--
100–199 cells/mm^3^	167 (31.9%)	126 (33.0%)	155 (47.6%)
200–299 cells/mm^3^	102 (19.5%)	74 (19.4%)	90 (27.6%)
≥300 cells/mm^3^	97 (18.6%)	70 (18.3%)	81 (24.9%)
Missing	88	--	
Viral load after 12 months of treatment			
Median VL (IQR) copies/mL	39.9 (39.9–39.9)	39.9 (39.9–39.9)	39.9 (39.9–39.9)
<40 copies/mL	430 (81.1%)	382 (100.0%)	270 (82.8%)
40–999 copies/mL	71 (13.4%)	--	40 (12.3%)
1,000–9,999 copies/mL	16 (3.0%)	--	10 (3.1%)
≥10,000 copies/mL	13 (2.5%)	--	6 (1.8%)
Missing viral load	80	--	--
Outcome at 12 months follow-up			
Died	35 (5.7%)	--	--
Defaulted	13 (2.1%)	--	--
Transferred out	15 (2.5%)	--	--
Retained in care, undetectable viral load	430 (70.5%)	382 (100.0%)	270 (82.8%)
Retained in care, detectable viral load	100 (16.4%)	--	56 (17.2%)
Retained in care but no viral load measured done at 12 mo	17 (2.8%)	--	--
Median time (IQR) between baseline & 12 mo CD4 count in days	386 (372–413)	386 (372–412)	391 (372–411)
Median time (IQR) between start of cART & 12 mo VL in days	366 (361–374)	366 (361–374)	366 (361–374)
Median time (IQR) between start of cART & 12 mo CD4 count in days	364 (356–374)	364 (357–373)	364 (356–373)

Numbers in Table 3 are N (%), unless mentioned otherwise. HIV Human immunodeficiency virus; cART combination antiretroviral treatment; IQR Inter-quartile range; N Number; mo month; VL Viral load.

Of the 382 patients in Nested Cohort 1, 112 (29%) had a rise in CD4 cell count <100 cells/mm^3^ between baseline and 12-month measurements. In bivariate analysis, only older age was significantly associated with an immunological discordant response (Table 4). In multivariable analysis, older age (*P* = 0.002), and having a longer travel time to the clinic (*P* = 0.01) were significantly associated with an immunological discordant response. Those from the Kayonza/Kirehe region appeared to be less likely to have a discordant treatment response; however, this difference was not statistically significant (*P* = 0.09). There was no association between baseline CD4 count and an immunological discordant response (*P* = 0.9), and none of the other demographic or health factors were associated (Table 4).

**Table 4:** Analysis of determinants of immunological discordant treatment responses (CD4 count rise <100 cells/mm^3^ despite complete virological suppression) in Nested Cohort 1, and of virological discordant treatment responses (CD4 count rise ≥100 cells/mm^3^ but incomplete virological suppression, i.e. VL≥40 copies/mL) in Nested Cohort 2, Rwanda, 2007–2008.

	Immunological discordant response (Nested Cohort 1)					Virological discordant response (Nested Cohort 2)				
		Bivariate analysis		Multivariable analysis			Bivariate analysis		Multivariable analysis	
			n/N (%)	OR (95% CI)	P	aOR (95% CI)	P	n/N (%)	OR (95% CI)	P	aOR (95% CI)	P
	112/382 (29.3%)					56/326 (17.2%)				
Age-group			<0.001		0.002			0.36		0.34
20–34 years	15/98 (15.3%)	1		1		18/101 (17.8%)	1		1	
35–44 years	47/159 29.6%)	2.3 (1.2–4.4)		2.4 (1.2–4.6)		18/130 (13.9%)	0.7 (0.4–1.5)		0.6 (0.3–1.3)	
≥45 years	50/125 (40.0%)	3.7 (1.9–7.1)		3.9 (2.0–7.8)		20/95 (21.1%)	1.2 (0.6–2.5)		1.0 (0.5–2.2)	
Sex			0.32		0.44			0.03		0.05
Male	45/139 (32.4%)	1		1		28/122 (23.0%)	1		1	
Female	67/243 (27.6%)	0.8 (0.5–1.3)		0.8 (0.5–1.3)		28/204 (13.7%)	0.5 (0.3–1.0)		0.5(0.3–1.0)	
Marital status			0.56					0.97		
Single	3/16 (18.8%)	0.6 (0.2–2.1)				3/16 (18.8%)	1.1 (0.3–4.0)			
Married/cohabiting	62/221 (28.1%)	1				34/193 (17.6%)	1			
Divorced/separated	11/38 (29.0%)	1.0 (0.5–2.2)				6/33 (18.2%)	1.0 (0.4–2.7)			
Widowed	36/107 (33.6%)	1.3 (0.8–2.1)				13/84 (15.5%)	0.9 (0.4–1.7)			
Literacy			0.54					0.27		
Unable to read	41/131 (31.3%)	1				23/113 (20.4%)	1			
Able to read	71/251 (28.3%)	0.9 (0.5–1.4)				33/213 (15.5%)	0.7 (0.4–1.3)			
CD4 count at baseline			0.85		0.87			0.29		0.25
<100 cells/mm^3^	16/53 (30.2%)	1		1		6/43 (14.0%)	1		1	
100–199 cells/mm^3^	22/82 (26.8%)	0.8 (0.4–1.8)		1.1 (0.5–2.1)		18/78 (23.1%)	1.9 (0.7–5.1)		2.1 (0.7–5.8)	
200–350 cells/mm^3^	74/247 (30.0%)	1.0 (0.5–1.9)		1.1 (0.6–2.2)		32/205 (15.6%)	1.1 (0.4–2.9)		1.3 (0.5–3.3)	
WHO Stage at baseline			0.66					0.92		
1 and 2	58/204 (28.4%)	1				30/176 (17.1%)	1			
3 and 4	54/177 (30.5%)	1.1 (0.7–1.7)				26/149 (17.5%)	1.0 (0.6–1.8)			
BMI at baseline			0.52					0.92		
<18.5 kg/m^2^	30/87 (34.5%)	1.4 (0.8–2.3)				12/69 (17.4%)	1.0 (0.5–2.1)			
18.5–24.9 kg/m^2^	71/256 (27.7%)	1				38/223 (17.0%)	1			
≥25 kg/m^2^	11/37 (29.7%)	1.1 (0.5–2.3)				6/32 (18.8%)	1.1 (0.4–3.0)			
On TB treatment at start cART				0.40							
No	109/366 (29.8%)	1				56/313 (17.9%)	N.A.			
Yes	3/15 (20.0%)	0.6 (0.2–2.1)				0/12 (0.0%)				
Regimen with d4T			0.26					0.78		
No	39/117 (33.3%)	1				17/95 (17.9%)	1			
Yes	73/264 (27.7%)	0.8 (0.5–1.2)				38/229 (16.6%)	0.9 (0.5–1.7)			
Regimen with NVP			0.70					0.96		
No	9/34 (26.5%)	1				5/30 (16.7%)	1			
Yes	103/347 (29.7%)	1.2 (0.5-(2.6)				50/294 (17.0%)	1.0 (0.4–2.8)			
Travel time to clinic			0.07		0.01			0.28		
<30 minutes	23/78 (29.5%)	1		1		7/62 (11.3%)	1			
Between 30 & 60 min.	27/93 (29.0%)	1.0 (0.5–1.9)		1.0 (0.5–2.0)		17/83 (20.5%)	2.0 (0.8–5.2)			
Between 1 & 2 hrs	28/124 (22.6%)	0.7 (0.4–1.3)		0.8 (0.4–1.5)		17/113 (15.0%)	1.4 (0.5–3.6)			
> 2 hrs	34/86 (39.5%)	1.6 (0.8–3.0)		2.4 (1.1–5.5)		15/67 (22.4%)	2.3 (0.9–6.0)			
Region			0.61		0.09			0.56		0.79
Ruhengeri	60/197 (30.5%)	1		1		26/163 (16.0%)	1		1	
Kayonza/Kirehe	52/185 (28.1%)	0.9 (0.6–1.4)		0.6 (0.3–1.1)		30/163 (18.4%)	1.2 (0.7–2.1)		1.1 (0.6–2.0)	

HIV human immunodeficiency virus; cART combination antiretroviral treatment; BMI body mass index; OR Odds ratio; aOR adjusted odds ratio; CI Confidence interval; TB tuberculosis; WHO World Health Organization; d4T stavudine; NVP nevirapine; min minutes; hrs hours. Complete virological suppression = viral load <40 copies/mL after 12 months of cART.

Of the 382 patients included in the analysis of an immunological discordant treatment response, 326 (85%) had a 3-month adherence measurement (assessed at a median of 85 days (IQR 82–98) after start of cART). In bivariate analysis good adherence at 3 months was not significantly associated with a lower risk for a discordant treatment response at 12 months (OR = 0.5, 95%CI 0.3–1.2, *P =* 0.1). When adherence was added to the multivariable model obtained in the primary analysis, good adherence was also not significantly associated (aOR = 0.5, 95%CI 0.2–1.1, *P* = 0.08). The effect size of all other variables in the model only changed marginally (data not shown). A similar analysis was done for adherence at 12 months; 12-months adherence was not associated with a discordant treatment response (*P* = 0.7), nor did it change the effect of the other variables in the model.

### Analysis of virological discordant treatment responses: Nested Cohort 2

Table 2 shows the baseline characteristics of Nested Cohort 2, alongside the characteristics of the full cohort and those of Nested Cohort 1. The median age (interquartile range [IQR]) was 39 (33–46) years and 63% of patients were female. The median (IQR) CD4 count at baseline was 239 (155–294) cells/mm^3^. Forty-six percent (149/326) of patients were in WHO stage 3 or 4 at the start of the treatment. The median time between baseline CD4 count and the date of start of cART was 23 (9–42) days.

Table 3 shows the follow-up data of Nested Cohort 2, next to the data of the full cohort and of Nested Cohort 1 for comparison. The median (IQR) CD4 count at 12 months was 454 (362–541) cells/mm^3^ and the median increase between the baseline CD4 count and the 12-month CD4 count was 206 (156–297) cells/mm^3^; by definition, all had an increase in CD4 count of at least 100 cells/mm^3^.

Of the 326 patients in Nested Cohort 2, 56 (17%) did not have full virological suppression. In bivariate analysis women had a lower risk for a virological discordant response (OR = 0.5, 95%CI 0.3–1.0; *P* = 0.03), but none of the other variables were associated with a virological discordant response (Table 4). In multivariable analysis, including (a priori) age, sex, baseline CD4 count and region, none of the variables were significantly associated with a discordant virological response (Table 4), although female sex was of borderline significance (*P* = 0.05).

Sixteen of the 326 patients in Nested Cohort 2 (5%) had a VL of 1,000 copies/mL or above. We also assessed determinants of a discordant treatment response defined in this less strict way. No significant determinants of such a discordant treatment response were identified, but power was limited.

An adherence measurement at 3 months was available for 278 (85%). In bivariate analysis good adherence at 3 months was not associated with a virological discordant treatment response (OR = 0.7, 95%CI 0.3–2.1, *P =* 0.5). When we added adherence to the previously obtained multivariable model, good adherence at 3 months was not a significant determinant of this type of discordant treatment response (aOR = 0.7, 95%CI 0.2–2.2; *P* = 0.5); the effect of the other variables did not change substantially (data not shown). A similar analysis was done for adherence at 12 months; 12-months good adherence was associated with a non-significantly decreased likelihood for a discordant treatment response (OR = 0.6, 95%CI 0.3–1.3, *P =* 0.2). When the 12-month adherence was added to the multivariable model, it did not change the effect of the other variables in the model substantially, but good adherence was associated with lower odds of a discordant treatment response (aOR = 0.5, 95%CI 0.2–1.2, *P =* 0.1).

## Discussion

Among patients with a CD4 rise less than 100 cells/mm^3^, 20% had a detectable VL, but only 5% had virological failure defined as VL>1,000 copies/mL. Conversely, 17% of patients with a CD4 rise of 100 cells/mm^3^ or more did not have full virological suppression. Together, these results indicate the poor predictive value of a rise in CD4 count for virological suppression and underscore the importance of viral load testing for treatment monitoring. Discordant treatment responses (i.e., the absence of an adequate immunological response despite an undetectable VL or vice versa) were observed in 36% of cART-naïve HIV patients in Rwanda, 12 months after the start of cART. Older age and long travel distance to the clinic were associated with an immunological discordant treatment response.

The frequency of immunological discordant treatment response in this study population was higher than that observed in several other studies, where the proportion ranged between 9% and 28%.[1,6–8,10,24–27] Immunological discordant treatment response remains an unsolved challenge to physicians involved in long-term HIV care and treatment. In agreement with most other studies we found that older age was a significant risk factor for immunological discordant treatment responses.[1,6,7,9,10,15] The degree of immune restoration is dependent on the thymic function; as thymic function decreases with age,[9,24,25] this may explain why older people are at higher risk of incomplete immune restoration. All our patients were on a regimen including a non-nucleoside reverse transcriptase inhibitor (NNRTI). NNRTIs are known to be associated with a lower CD4 increase after the start of cART,[26] with particular evidence of this effect for zidovudine.[28,29] In our study, neither type of NNRTI (zidovudine or stavudine) was associated with either type of discordant treatment response.

Most studies found that low VL at start of cART was associated with immunological discordant treatment responses,[6,7,10,15] but as we did not have baseline VL available, we could not assess this in our cohort. Regarding the effect of baseline or nadir CD4 count, studies have provided contradictory results. Some found that a low baseline or nadir CD4 count was predictive of immunological discordant treatment responses,[1,6,15] while others found the reverse,[7,10] and some studies found no association between baseline CD4 count and immunological discordant treatment response.[9,11] The remarkable finding that a higher baseline CD4 count is a significant predictor of a discordant treatment response is probably due to regression to the mean. Due to random variation in the measurement of CD4 counts and due to individual variation of CD4 counts, those with the highest CD4 counts are bound to have lower CD4 counts upon subsequent measurements even in the absence of any intervention.[8,27] In our cohort we did not observe any effect of baseline CD4 count on discordant treatment responses. Evolving definitions of discordant treatment response,[23,30,31] and variation in the definitions used across studies may in part explain discrepancies in findings as well as variability in the frequency of this condition.[1,7,11]

We also found that an immunological discordant treatment response was more common in patients who had to travel more than two hours to the clinic. This was not explained by a lower adherence among them and could be a chance finding. In a previously published analysis of this cohort,[32] time to clinic was not associated with retention with VL suppression. Future investigations could further explore the association between travel time to health clinics, and treatment outcomes and discordant responses. We did not find that TB treatment at the start of cART predicted an immunological discordant response though only a small number of patients had TB.

Discordant virological treatment response—incomplete suppression of viral replication but a good immunological response—was observed in 17% of patients with a CD4 rise of 100 cells/mm^3^ or more. We could not identify any independent determinants of such discordant treatment response, although female sex was associated with lower odds of discordance at *P* = 0.03. The interpretation of these data is not straightforward, as the VL might have become detectable just because of a brief interruption of therapy. This is supported by the analysis in which adherence at 12-months was included; poor adherence at that time point was significantly associated with a discordant response. Ongoing screening for suboptimal adherence and targeted, intensive counselling may improve virological responses and prevent acquired resistance for individuals with inconsistent adherence. Repeat CD4 count and viral load assessments in this group will be critical to distinguishing between incomplete viral suppression due to suboptimal adherence and that due to drug resistance. Most of the patients with detectable VL (71%; 40/56) had VL <1,000 copies/ml. We only had a single VL measurement, so we could not distinguish between transient viremia, also referred to as “viral blips,” or full-blown prolonged viremia associated with poor adherence or resistance. Nevertheless, these data underscore that clinicians should be equally careful not to switch cART too early because of transient viremia, especially when viral blips do not exceed 400 copies/mL. In this case, adherence counselling should be considered, as viral blips are often associated with short-term lapses in adherence rather than resistance.[33,34]

Because this was a secondary analysis of a prospective study of the first year of cART, and viral load was conducted one time at the end of study follow-up, we lacked information regarding physician’s decision-making patterns in the absence of viral load monitoring. Although Rwanda has since implemented routine viral load monitoring, many settings still lack access to this important tool. In such programs limited CD4 count rises might be erroneously interpreted as treatment failure and patients may subsequently be switched to second line regimens. In our study only 5% of patients with a limited CD4 rise after 12 months had virological failure (VL ≥1,000 copies/mL). This suggests that in most such cases the (costly) treatment change is unnecessary. VL tests are needed to avoid this; in public health clinics such tests could differentiate between ART failure and a (less threatening) discordant treatment response. Nevertheless, both discordant treatment responses are associated with poorer outcomes,[6,8,9,11] and should, if diagnosed, be regarded as a danger sign to the clinician.

### Limitations

Our study has several limitations. We did not have baseline VL measurements and for the definition of viral success we relied on a single VL determination approximately 12 months after the start of cART. If that single VL determination was not representative of the VL over time in a particular patient, misclassification might have occurred, regarding some patients as virological responders who in fact were not having a complete virological response to therapy and vice versa. Furthermore, a single VL measurement of more than 1,000 copies/mL does not infallibly identify the development of viral resistance to the antiretroviral therapy used. A high VL can be caused by temporary treatment interruptions, which is usually quickly and fully re-suppressed after re-initiation of cART. Also, we relied on only one CD4 count taken at approximately 12 months. Daily fluctuations in CD4 counts and fluctuations induced by opportunistic infections are common, so this may have led to another misclassification, in two directions: regarding some persons unfairly as immunological non-responders and others unfairly as immunological responders. So long as the misclassification was not associated with the predictors under study, we would expect it to lead to underestimations of the true associations. This misclassification might have reduced power to detect significant associations with baseline variables.

The size of the cohort and the small number of events (especially that of virological discordance) meant that the power of this study to identify significant determinants was limited, and this may be the reason we could not confirm some of the associations that earlier studies had found. Lastly, there may be other factors that were not measured in this cohort (i.e., drug substitutions, smoking) that predict a discordant response.

## Conclusion

Discordant treatment response are relatively common and signal a patient who is vulnerable to negative health outcomes and may benefit from intensified adherence counseling and repeat laboratory monitoring over the short term. When VL monitoring is not done (as is the case in many ART programs in sub-Saharan African countries), a limited increase of the CD4 count after 12 months could be misinterpreted as a (virological) treatment failure and lead to unnecessary changes to more expensive second-line ART regimens. Development and implementation of low-cost point of care VL tests, coupled with ongoing adherence outcomes and a strong drug supply chain, will likely help optimize cART outcomes. Future research should be conducted to further understand the long-term consequences of discordant immunological treatment responses.
